# 906. Impact of Accelerate Rapid Diagnostic Technology on Time to Optimal Antimicrobial Therapy in Gram Negative Bloodstream Infection

**DOI:** 10.1093/ofid/ofac492.751

**Published:** 2022-12-15

**Authors:** Hye Lim Jung, Caroline Jozefczyk, Carmen M Faulkner-Fennell, Rhett Shirley, John Schrank

**Affiliations:** Prisma Health-Upstate, Greenville, South Carolina; Prisma Health - Upstate, Greenville, South Carolina; Prisma Health-Upstate, Greenville, South Carolina; Prisma Health Infectious Diseases - Greenville, Greenville, South Carolina; Prisma Health Infectious Diseases - Greenville, Greenville, South Carolina

## Abstract

**Background:**

Previous studies at our institution evaluating rapid diagnostic testing (RDT) for blood culture identification (BCID) did not show significant differences in time to optimal antimicrobial therapy for Gram negative bloodstream infections (GNBSI). Recent studies of Accelerate Pheno® system (Accelerate-PS) for blood culture identification and susceptibility have shown significant reduction in time to optimal antimicrobial therapy. This study evaluated the impact of Accelerate-PS compared to BCID RDT on time to optimal antimicrobial therapy for GNBSI.

**Methods:**

A single center, retrospective, cohort study included adult patients with GNBSI from February 2017 to January 2018 (pre-implementation) and February 2020 to January 2021 (post-implementation). The primary outcome was time to optimal antimicrobial therapy for GNBSI, defined as time from positive blood cultures to time patient received optimal antimicrobial therapy. Secondary outcomes include duration of therapy, rate of antimicrobial-related adverse effects, hospital length of stay, in-hospital mortality, and infection-related readmission.

**Results:**

The final cohort included 190 patients in pre-implementation group and 179 patients in post-implementation group. *Escherichia coli* and *Klebsiella* species were the most common pathogens and urinary tract was the most common source of bacteremia in both groups. More patients in the pre-implementation group had congestive heart failure while more patients in the post-implementation group had peripheral vascular disease. Patients in the post-implementation group had higher Pitt bacteremia scores (1.05 vs. 1.37, P=0.017). Patients in the post-implementation group had significantly shorter time to optimal therapy (mean 60.62 hours vs. 20.17 hours, P< 0.001) and shorter duration of therapy (mean 366.56 vs 310.24 hours, P < 0.001) than in the pre-implementation group. There were no differences in mortality or readmission at 30-days.

**Conclusion:**

The results of this study indicate that incorporation of Accelerate-PS into microbiology lab workflow significantly reduces time to optimal antimicrobial therapy for GNBSI. Rapid diagnostic testing is a vital component of a robust antimicrobial stewardship program.

**Disclosures:**

**John Schrank, MD**, Gilead: Speaker's Bureau.

